# Pulmonary veins isolation to retard pacemaker implantation

**DOI:** 10.1002/ccr3.1865

**Published:** 2018-10-30

**Authors:** Laurence van der Haert, Dominique Blommaert

**Affiliations:** ^1^ Cardiology Centre Hospitalier Universitaire Dinant/Godinne Yvoir Belgium

**Keywords:** mental retardation, pulmonary veins isolation, scoliosis, sick sinus syndrome, syndromic form

## Abstract

Sick sinus syndrome is uncommon in children, and syndromic forms are rare. Some forms of sick sinus syndrome like the bradycardia‐tachycardia type could be managed by a radiofrequency ablation, even in young children, and could be helpful to delay the implantation of a pacemaker.

## 1 INTRODUCTION

Sick sinus syndrome is most often seen in elderly, with multiple etiologies, including degenerative fibrosis of the sinoatrial node or remodeling of this node in heart failure and atrial fibrillation.^1^ In children, it is an uncommon disease that is mostly associated with other cardiac abnormalities, mainly after cardiac surgery, but also in children with unoperated congenital heart disease or in children with viral myocarditis.^2^ Syndromic forms of sick sinus syndrome are rare. We present the case of a young patient with a sick sinus syndrome, mental retardation and severe scoliosis, association that has rarely been described.

## 2 CASE PRESENTATION

**Figure 1:**
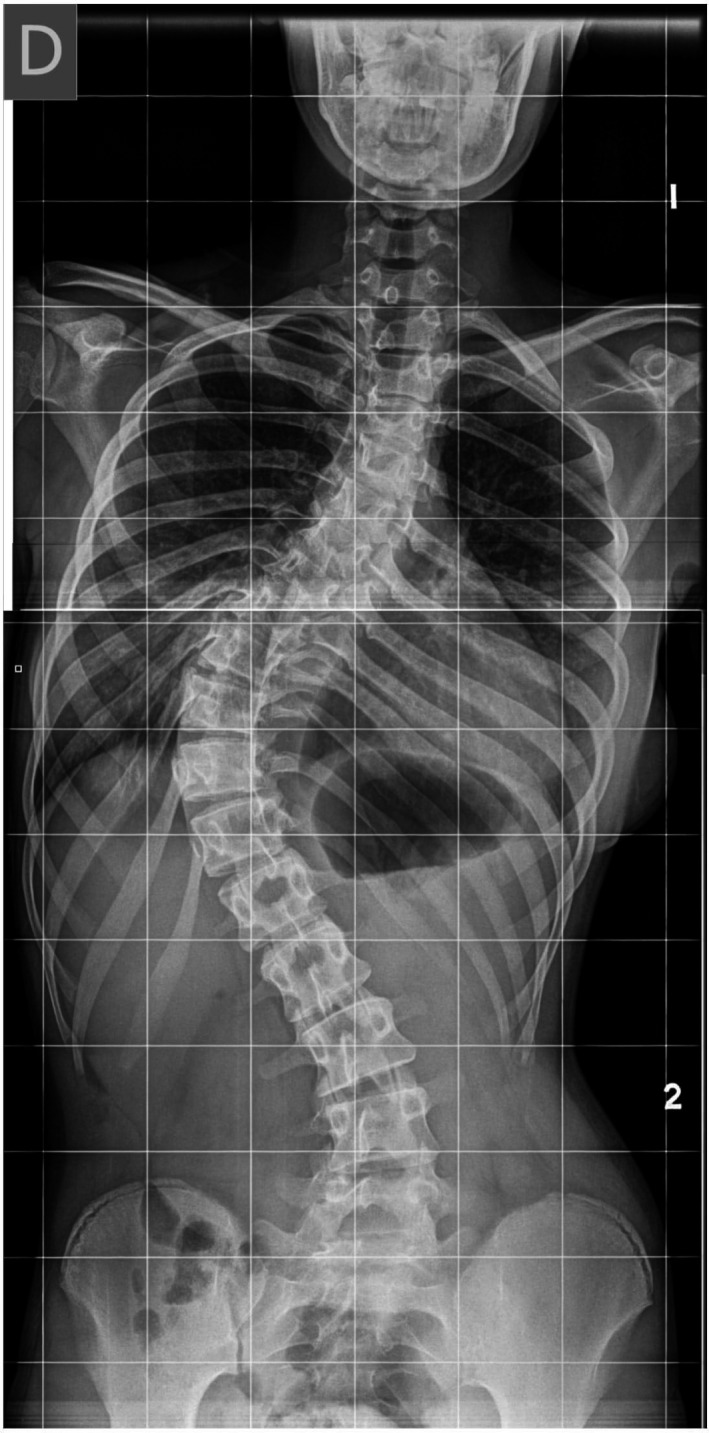
Radiography showing the dorso‐lumbar scoliosis, before correction

A 15‐year‐old female patient was first seen in our service in 2012 for a second opinion regarding the treatment of her cardiac arrhythmias. Her medical history includes mental retardation, delay of psychomotor development, and a progressive dorso‐lumbar scoliosis (Figure 1) for which she underwent an orthopedic correction at the age of 15 years, 5 months before she was first seen in our service. Her family's medical history is unremarkable. She was being followed in another institution for a sinus bradycardia discovered during the neonatal period. Since the age of 14 years old, she also presented paroxysmal atrial fibrillation. To allow an intensification of her antiarrhythmic treatment, a pacemaker implantation was one of the options proposed in the other institution.

Despite her mental retardation, the patient could express that she had no heart related symptoms such as dyspnea, palpitations, or chest pain, and had no limitations in her activities of daily living, such as walking or doing some indoor cycling. There was no history of syncope. This was confirmed by her parents, who were most of the time with her.

**Figure 2:**
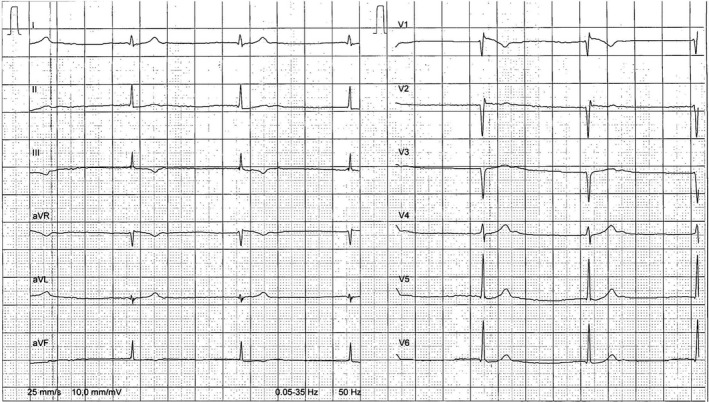
12‐lead electrocardiogram showing a junctional rhythm with narrow QRS complexes

**Figure 3:**
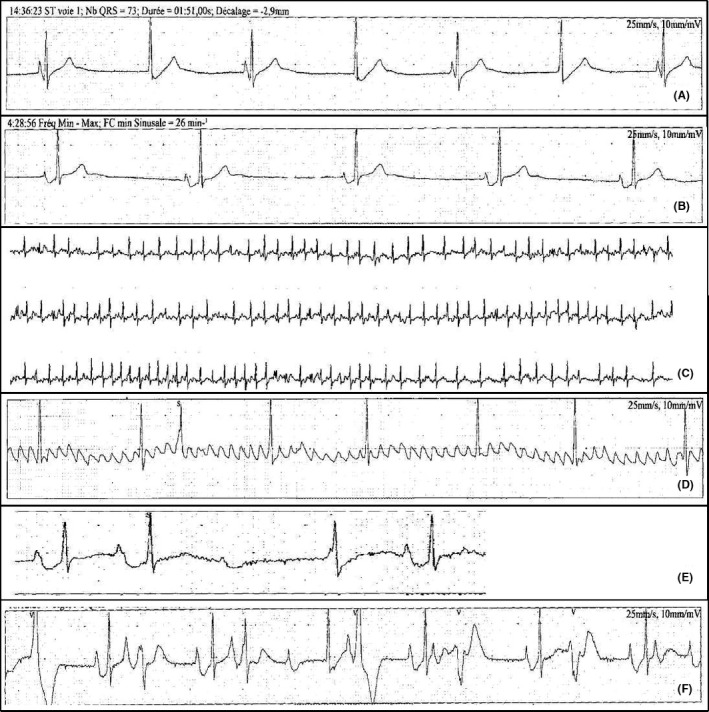
A, Diurnal sinus bradycardia alternating with junctional rhythm. B, Nocturnal sinus bradycardia. C, Atrial fibrillation with rapid ventricular response. D, Organized atrial fibrillation. E, Wenckebach atrioventricular block. F, Polymorphic premature ventricular contractions

Her 12‐lead electrocardiogram showed a junctional rhythm, with narrow QRS complexes (Figure 2). A 6‐day holter monitor revealed sinus bradycardia, junctional rhythm, sinus pauses, short episodes of Wenckebach atrioventricular block, 20% of atrial fibrillation, atrial flutter, and premature ventricular contractions with episodes of bigeminy (Figure 3A‐F). Her echocardiography did not detect any abnormality, without left atrial dilatation. The patient supported a load of 120 Watt at her exercise test that allowed reaching a heart rate of 132 beats per minute (65% of the maximum heart rate).

**Figure 4:**
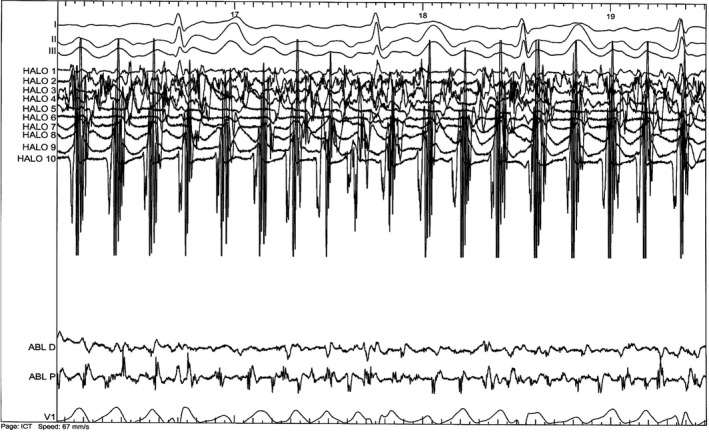
Atrial fibrillation with a cycle length of 110 ms in the left pulmonary veins

**Figure 5:**
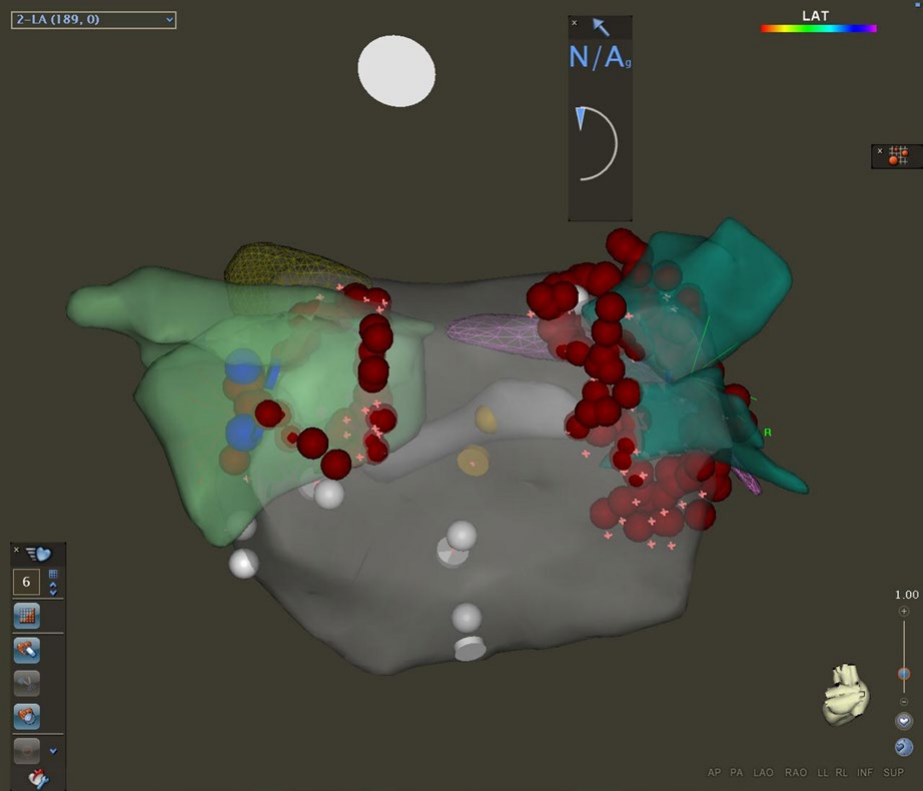
Isolation of the pulmonary veins using a point‐by‐point technique, ablating the left and right ipsilateral pulmonary veins circumferentially

An electrophysiological study was conducted in our institution, showing atrioventricular conduction abnormalities with episodes of Wenckebach atrioventricular block. AH interval was measured at 76 ms and HV interval at 53 ms. There was a duality of the atrioventricular conduction without induction of atrioventricular nodal reentrant tachycardia, with or without isoprenaline. The QTc interval was prolonged at 540 ms after isoprenaline infusion. Atrial fibrillation started at the transseptal puncture. The cycle length was 196 ms in the left atrial appendage and in the right atrium, 150 ms in the right pulmonary veins, and 110 ms in the left pulmonary veins (Figure 4). The left and right ipsilateral pulmonary veins were circumferentially ablated using a point‐by‐point technique (Figure 5). Sinus rhythm was restored during the antrum isolation of the left pulmonary veins. At the positioning of the lasso in the right pulmonary veins, an atypical left atrial flutter started with restoring sinus rhythm during the isolation of these veins.

No mutations associated with sick sinus syndrome (LMNA, SCN5A, CACNA1D, GJA5, HCN4, MYH6, PRKAG2, SCN1B, TBX5, TRPM4, NKX2‐5, TBX3, and WWTR1) or long QT syndrome (KCNQ1 and KCNH2) were found in the DNA tests performed in 2013.

The patient is followed once a year in our service. She was seen for the last time, for her sixth follow‐up consultation in March 2018, five years after the isolation of the pulmonary veins. She takes no medication and is still asymptomatic of her sick sinus syndrome. The 6‐day holter monitor showed sinus bradycardia, junctional rhythm, sinus pauses, and premature supraventricular contractions, but no atrial fibrillation, atrial flutter and almost no premature ventricular contractions.

## 3 DISCUSSION

We presented the case of a syndromic form of sick sinus syndrome, associating bradycardia‐tachycardia syndrome, mental retardation, delay of psychomotor development, and dorso‐lumbar scoliosis. To our knowledge, this is the second time that this association has been described. Chinnamuthu Murugesan et al presented a similar case of an 18‐year‐old man withcongenital anomalies/mental retardation syndrome associated with bradycardia‐tachycardia syndrome. His chromosomal study was unremarkable, but they did not look for specific DNA mutations.^3^ Other associations have been described, with bone malformation caused by a Connexin‐45 mutation,^4^ with myotonic dystrophy,^5^ or with CREST syndrome.^6^ Sick sinus syndrome has also been described in patients with Rett syndrome,^7^ characterized by a stagnation and regression of psychomotor development after the age of 6‐24 months, resulting in severe dementia, loss of speech, loss of social response and loss of purposeful hand use. Common features are seizures, episodic hyperpnea, scoliosis, spasticity, and vasomotor disturbances of lower limbs.^8^ Our patient presents some of these symptoms (mental retardation and scoliosis) but most are not present. Her psychomotor development was delayed but eventually completed, and she can follow a normal conversation and has no problems with the use of her hands. Therefore, although we did not look for specific mutations that are associated with this disease, we can reasonably exclude the diagnosis.

In patients with a structurally normal heart, sick sinus syndrome is mostly associated with channelopathies. The most frequent reported mutations are in the SCN5A gene and in the HCN4 gene.^9^, ^10^, ^11^, ^12^ None of these or other mutations associated with sick sinus syndrome were found in our patient but it is likely that other mutations will be discovered in the future.

Sick sinus syndrome can be caused by a number of intrinsic or extrinsic factors. These lead to multiple atrial abnormalities, with both structural and electrophysiological remodeling of the atria. Patients with sinus node dysfunction present left atrial enlargement and regions of low voltage amplitude and spontaneous scarring, suggesting loss of automatic pacemaker cells in these regions.^13^ At a molecular level, sick sinus syndrome is the result of complex alterations in multiple processes that contribute to the automaticity of the sinoatrial node.^14^ Multiple studies have shown a relationship between sinus node dysfunction and atrial fibrillation. Sinus bradycardia in sick sinus syndrome can strongly facilitate the conditions for atrial fibrillation occurrence by both increasing the atrial ectopy and dispersion of the refractoriness.^15^ On the other hand, Chang et al showed that in patients with atrial fibrillation, regional atrial remodeling near the sinus node area was associated with prolonged sinus node recovery time, a longer right atrial activation time, a lower voltage of the sinoatrial node area, and a slower conduction velocity, characteristics of sinus node dysfunction.^16^ In a study from Hocini et al, ablation to cure atrial fibrillation resulted in a reverse modeling of sinus node function with an increase of the mean heart rate, maximal heart rate, and heart rate range and a decrease in the corrected sinus node recovery time.^17^

The management of bradycardia‐tachycardia syndrome is challenging, because all medication that can help to control the episodes of tachycardia are likely to intensify the episodes of bradycardia. In our case, a pacemaker implantation was proposed to the patient even if she had never had an episode of syncope. Pacemaker implantation in children must be well thought through because, even if the risks at short term are very small, these children will be reoperated multiple times, with a high risk of device infection and associated infective endocarditis at long term. This is why we decided to propose an electrophysiological study with isolation of the pulmonary veins. The procedure was very effective, with a 5‐year follow‐up without atrial fibrillation, other supraventricular tachycardia, or atrioventricular conduction abnormalities andalmost no premature ventricular contractions, this without any medical treatment. It has been demonstrated that the autonomic ganglia at the pulmonary vein ostia play a role in the initiation and maintenance of atrial fibrillation.^18^ There is also evidence that left cardiac sympathetic denervation is associated with a reduction of ventricular events, for example in patients with long QT syndrome.^19^ Although there is no evidence for it in the literature, the ablation of the autonomic ganglia during the isolation of the pulmonary veins could, in addition to the possible role in the absence of atrial fibrillation and flutter in the 5‐year follow‐up of our patient, also play a role in the reduction of the premature ventricular complexes.

A pacemaker will probably be implanted at some point in this patient with persisting sinus bradycardia/junctional rhythm, but we prefer to wait as long as possible to avoid serious complications, all the more so as she was asymptomatic at her 5‐year follow‐up.

## 4 CONCLUSION

In conclusion, we reported a case of sick sinus syndrome in a patient with a structurally normal heart, associated with mental retardation and severe scoliosis. This association has been described only once before. No specific DNA mutations for sick sinus syndrome were found in our patient. We proposed a pulmonary veins isolation to treat the episodes of atrial fibrillation, which was very effective, to delay the implantation of a pacemaker in this young woman.

## CONFLICT OF INTEREST

None declared.

## AUTHOR CONTRIBUTION

LH: wrote the paper and wrote a review of the literature concerning sick sinus syndrome in children. DB: proposed an electrophysiological study with isolation of the pulmonary veins.
